# Pregnancy Loss and Risk of All-Cause Mortality in Chinese Women: Findings From the China Kadoorie Biobank

**DOI:** 10.3389/ijph.2023.1605429

**Published:** 2023-04-14

**Authors:** Li Jiang, Sha Huang, Jia Yi Hee, Yiqian Xin, Siyu Zou, Kun Tang

**Affiliations:** ^1^ Centre for Global Child Health, The Hospital for Sick Children, Toronto, ON, Canada; ^2^ Vanke School of Public Health, Tsinghua University, Beijing, China; ^3^ Duke Global Health Institute, Duke University, Durham, NC, United States

**Keywords:** stillbirth, all-cause mortality, spontaneous abortion, pregnancy loss, maternal health

## Abstract

**Objectives:** Pregnancy loss is a common obstetric complication that may be associated with maternal mortality. However, evidence is sparse and inconsistent. This study aims to investigate the association between pregnancy loss with the risk of all-cause mortality among Chinese women.

**Methods:** Data on 299,582 women aged 30–79 years old from the China Kadoorie Biobank were used. Cox proportional hazard regression was conducted to investigate the association between the occurrence of pregnancy loss and all-cause mortality.

**Results:** Two or more pregnancy losses was associated with long-term all-cause mortality (adjusted hazard ratio (aHR) of 1.10, 95% CI: 1.03–1.18). Specifically, more than one spontaneous abortion or stillbirth was associated with long-term all-cause mortality (aHR 1.10, 95% CI: 1.01–1.21 and 1.14, 95% CI: 1.04–1.25, respectively). When stratified by the presence of cardiovascular disease or diabetes, as well as age at baseline, two or more pregnancy losses in women aged ≥50 diagnosed with cardiovascular disease (aHR 1.32, 95% CI: 1.18–1.48) or diabetes (aHR 1.30, 95% CI: 1.06–1.60) was associated with all-cause mortality.

**Conclusion:** Recurrent pregnancy loss, in particular two or more spontaneous abortions and stillbirths were associated with increased risk of all-cause mortality. The associations between recurrent pregnancy losses and all-cause mortality were more pronounced in women aged ≥50 with cardiovascular disease or diabetes at baseline.

## Introduction

Pregnancy loss describing spontaneous abortion or stillbirth is a common obstetric complication that occurs in approximately every 1 in 4 pregnancies (1,2). Recurrent pregnancy loss, which affects approximately 1%–2% of women is defined as the loss of two or more pregnancies (3). Spontaneous abortion, also referred to as miscarriage, typically occurs before the 20th week of pregnancy (4), while stillbirth typically occurs after the 28th week of pregnancy but before or during birth (5). However, many developed countries continue to utilized the threshold for stillbirth after the 20th or 22nd week of pregnancy (5,6). Due to the varying definitions utilized, the prevalence of pregnancy loss varies from 10% to 24% of all clinically-confirmed pregnancies (7–9) and approximately one-third of all pregnancies (9,10).

The influence of pregnancy loss on women may extend beyond the perinatal period. Studies have demonstrated that pregnancy loss may share common risk factors with certain non-communicable diseases (10,11). A history of pregnancy loss have been associated with long-term adverse health outcomes, including cardiovascular diseases (12–16) and diabetes (17–20). Furthermore, 10%–15% of women with two or more recurrent spontaneous abortion have been diagnosed with antiphospholipid syndrome (APS) (21,22). APS may facilitate the development of severe complications, such as venous thromboembolism and stroke, and may therefore greatly impact quality of life and future health (23,24). Stillbirth have also been associated with APS (25).

The establishment of an association between pregnancy loss and chronic disease suggests that pregnancy loss may have long-lasting implications on overall maternal health. However, little is known about the association between pregnancy loss with the risk of mortality, particularly in low- and middle-income countries, as well as if type of pregnancy loss (e.g., spontaneous abortion and stillbirth) contributes similar risks. To date, no prospective studies conducted in China have assessed the relationship between pregnancy loss and all-cause mortality. Therefore, data from a large-scale prospective cohort will be used to investigate the association between pregnancy loss, including spontaneous abortion and stillbirth, with all-cause-mortality in Chinese women, stratified by the presence of cardiovascular disease and diabetes, as well as sociodemographic and lifestyle factors.

## Methods

### Study Settings and Participants

The present study utilizes data from the China Kadoorie Biobank (CKB), a prospective database, on 302,510 women aged between 30 and 79 years that were recruited between 2004 and 2008 from 10 geographically defined regions of China. From an initial cohort of 302,510 women, 2,881 women who reported never having been pregnant and 47 women who had missing data were excluded. The remaining 299,582 women were included in the final analysis. The study design, characteristics of participants, and survey methods of the CKB database have been previously described in detail elsewhere (26,27).

Briefly, data was collected through an interviewer-administered questionnaire, which included but was not limited to sociodemographic status, lifestyle factors, and personal and family medical history. Personal medical history consists of physician-diagnosed disease status. Respondents were asked: “Has a doctor ever told you that you had the following disease?” followed by a list of diseases including cardiovascular disease (coronary heart disease, hypertension, and stroke), and diabetes (27). The definitions of hypertension and diabetes included either the self-reported physician-diagnosed hypertension and diabetes or screening-detected hypertension and diabetes from the physical examination (28,29). In the present study, screen-detected hypertension was defined if participants had a measured diastolic blood pressure ≥90 mm Hg or systolic blood pressure ≥140 mm Hg. For self-reported diabetes, those diagnosed at an age below 30 years and currently being treated with insulin were considered as probable cases of type 1 diabetes, and were excluded at baseline enrollment. Screen-detected type 2 diabetes was defined as a random blood glucose level ≥11.1 mmol/L with a fasting time <8 h or a random blood glucose level ≥7.0 mmol/L with a fasting time ≥8 h or a fasting blood glucose ≥7.0 mmol/L. In addition, the median age of these participants at diabetes diagnosis were 54.00 (IQR: 48.00–60.00) years. The age of the majority of women at diabetes diagnosis was past the age of reproduction, therefore gestational diabetes may not occur. Factors relating to the reproductive history of women include age at menarche, parity, number of spontaneous abortions and stillbirths, menopausal status, and contraceptives use were also collected. Spontaneous abortion was defined as the loss of pregnancy naturally in the absence of elective medical or surgical measures to terminate the pregnancy. Stillbirth was classified as the death or loss of a fetus before or during delivery. Participants were considered to have pregnancy loss if they experienced spontaneous abortion or stillbirth. Anthropometric measurements: weight, height, and waist-hip circumference, were taken by trained technicians using standard protocol and procedures. Body mass index (BMI) was calculated as weight divided by the square of height (kg/m^2^). The physical activity level was measured by adding up metabolic equivalent tasks (METs) for daily work or leisure activities.

The CKB study was given ethics approval from the University of Oxford, Peking University, the China National Center for Disease Control and Prevention (CDC), and the institutional review boards of the local CDCs in the study areas. All participants have provided written informed consent according to the Declaration of Helsinki for participation (26).

### Follow-Up for Morbidity and Mortality

Death, including the cause of death, and health outcomes was collected periodically from baseline until 31 December 2016, *via* linkages with hospital records, national health registries, and social health insurance databases in the study areas. To minimize loss to follow-up, active follow-up involving visitations to the local community or direct contact with participants was performed annually (26). Fatal events entered into the CKB follow-up system were coded according to the 10th International Classification of Diseases (30). All records, including scanned images of original death certificates, were reviewed centrally by study clinicians blinded to baseline information (26). The outcome in analyses of this study was death from all causes. All participants were prospectively followed up, with a median follow-up of 10.20 years.

### Statistical Analysis

Baseline characteristics were presented as means (SD) for continuous variables and as percentages for categorical variables, stratified by the number of spontaneous abortions and stillbirths. Continuous variables were compared using the one-way analysis of variance (ANOVA) test for variables with normal distribution and the Kruskal-Wallis test for variables with skewed distribution. Categorical variables were compared using the chi-square test. The outcome was divided into two categories by the presence of all-cause mortality (yes or no). Cox proportional hazards regression was used to obtain the hazard ratio (HR) and 95% confidence intervals (CI) for the associations between pregnancy loss (total pregnancy loss, spontaneous abortion, and stillbirth) and all-cause mortality. Covariates were selected for inclusion in the models based on prior knowledge and published literature (13,19). Models were adjusted for age, region, BMI, education, annual household income, physical activity, smoking, alcohol consumption, cardiovascular disease, diabetes, number of livebirths and, where appropriate, number of spontaneous abortions, and stillbirths.

Subgroup analyses were also performed to obtain the adjusted HR (aHR) and 95% CI for the association between pregnancy loss and all-cause mortality by the presence of cardiovascular disease and diabetes, age (<50 or ≥50 years), BMI (<25 or ≥25 kg/m^2^), study region (rural or urban), level of education (elementary school and below, middle and high school, or university and above), annual household income (<20,000 or ≥20,000 yuan/year), and MET (<17 or ≥17 h/day). Statistical significance was set at *p* < 0.05 for all statistical analyses and were performed with SAS software package version 9.4 (SAS Institute Inc.).

## Results

### Characteristics of Study Participants

The characteristics of the participants stratified by the number of pregnancy loss are presented in Table 1. Among a total of 299,582 women who reported having ever been pregnant, 41,571 (13.88%) have experienced at least one pregnancy loss, of which 27,156 (9.06%) had a history of spontaneous abortion, and 17,041 (5.69%) had a history of stillbirth. Compared to women who have never had a pregnancy loss, women who have had a history of pregnancy loss were older (50.84 vs. one: 54.42, two or more: 57.86), had lower BMI (23.86 vs. one: 23.65, two or more: 23.32), were more likely to reside in rural regions (53.47% vs. one: 66.58%, two or more: 74.93%), were more likely to have educational levels elementary school and below (54.50% vs. one: 69.21%, two or more: 77.73%), have incomes of less than 2,500 yuan (2.91% vs. one: 3.88%, two or more: 5.35%), had lower MET hours (20.75 vs. one: 19.08%, two or more: 16.97%), were more likely to smoke (4.90% vs. one: 5.94%, two or more: 6.16%) but were less likely to drink alcohol (37.31% vs. one: 31.45%, two or more: 28.01%), and had more livebirth numbers (2.15 vs. one: 2.68, two or more: 3.06).

**TABLE 1:** Baseline characteristics of study participants by number of pregnancy losses (China, 2004–2008).

Characteristics	Total number of pregnancy loss^a^				Number of spontaneous abortion^b^				Number of stillbirth^a^			
	0	1	≥2	*p*-value	0	1	≥2	*p*-value	0	1	≥2	*p*-value
Total (n)	258,008	30,751	10,820		272,424	21,412	5,744		282,538	13,174	3,867	
Age, mean (SD), year	50.84 (10.24)	54.42 (10.84)	57.86 (11.23)	<0.01	51.20 (10.37)	53.75 (10.98)	55.18 (11.60)	<0.01	51.06 (10.34)	56.77 (10.47)	62.35 (9.49)	<0.01
BMI, mean (SD), kg/m^2^	23.86 (3.44)	23.65 (3.52)	23.32 (3.61)	<0.01	23.83 (3.45)	23.70 (3.52)	23.64 (3.64)	<0.01	23.85 (3.45)	23.40 (3.53)	22.80 (3.56)	<0.01
Socioeconomic factors												
Region, %				<0.01				<0.01				<0.01
Rural	53.47	66.58	74.93		54.22	68.42	72.72		54.73	67.52	77.92	
Urban	46.53	33.42	25.07		45.78	31.58	27.28		45.27	32.48	22.08	
Level of highest education, %				<0.01				<0.01				<0.01
Elementary school and below	54.50	69.21	77.73		55.72	67.56	70.28		55.53	75.6	88.98	
Middle and high school	40.79	28.45	20.84		39.74	29.89	27.58		39.94	22.78	10.68	
University and above	4.71	2.33	1.43		4.54	2.55	2.14		4.53	1.62	0.34	
Annual income, % (yuan/year)				<0.01				<0.01				<0.01
<2,500 yuan	2.91	3.88	5.35		2.99	3.92	4.94		3.01	4.09	6.36	
2.500–4,999 yuan	6.64	8.92	11.2		6.76	9.42	11.35		6.9	8.47	12.02	
5,000–9,999 yuan	19.19	21.89	22.35		19.15	23.67	24.76		19.63	18.59	19.39	
10,000–19,999 yuan	29.33	30.69	31.41		29.42	30.56	31.53		29.45	30.87	31.52	
20,000–34,999 yuan	24.76	21.51	19.67		24.72	20.02	17.39		24.27	24.46	21.39	
≥35,000 yuan	17.17	13.12	10.02		16.95	12.41	10.03		16.73	13.51	9.31	
Lifestyle factors												
Physical activity, mean (SD), (MET hours/day)	20.75 (12.85)	19.08 (12.19)	16.97 (11.37)	<0.01	20.55 (12.80)	19.52 (12.44)	18.53 (11.91)	<0.01	20.66 (12.82)	17.78 (11.47)	14.34 (9.77)	<0.01
Ever regular smoker, %	4.90	5.94	6.16	<0.01	4.96	5.81	6.53	<0.01	4.99	6.19	5.74	<0.01
Ever regular alcohol drinker, %	37.31	31.45	28.01	<0.01	36.51	34.87	35.2	<0.01	37.21	23.88	17.64	<0.01
Reproductive history												
Number of livebirths, mean (SD)	2.15 (1.29)	2.68 (1.46)	3.06 (1.64)	<0.01	2.18 (1.31)	2.71 (1.51)	2.92 (1.74)	<0.01	2.20 (1.33)	2.79 (1.44)	3.27 (1.49)	<0.01

a Missing value = 3.

b Missing value = 2.

BMI, body mass index; MET, metabolic equivalent task.

### Pregnancy Loss and All-Cause Mortality

The association between pregnancy loss with all-cause mortality is presented in Table 2 *.* Compared to women without pregnancy loss, women with a history of two or more pregnancy loss have higher all-cause mortality: aHR 1.10, 95% CI 1.03–1.18. Similarly, women with more than one spontaneous abortion or stillbirth have higher all-cause mortality: aHR 1.10, 95% CI 1.01–1.21 for two or more spontaneous abortions, and aHR 1.14, 95% CI 1.04–1.25 for two or more stillbirths, respectively. However, the associations were not statistically significant for women with one pregnancy loss, spontaneous abortion, or stillbirth.

**TABLE 2:** Cox proportional hazard ratios (95% confidence intervals) for all-cause mortality by pregnancy loss number (China, 2004–2008 for baseline characteristics and 2016 for all-cause mortality).

	Number of participants	Number of deaths	HR (95% CI)^a^	*p* for trend
Total pregnancy loss				0.02
0	258,008	10,435	1	
1	30,751	1905	1.01 (0.96, 1.06)	
≥2	10,820	1,094	1.10 (1.03, 1.18)**	
Spontaneous abortion				0.08
0	272,424	11,631	1	
1	21,412	1,298	0.99 (0.93, 1.05)	
≥2	5,744	505	1.10 (1.01, 1.21)*	
Stillbirth				0.03
0	282,538	11,940	1	
1	13,174	967	1.02 (0.95, 1.09)	
≥2	3,867	527	1.14 (1.04, 1.25)**	

^a^ Adjusted for age, region, BMI, level of highest education, annual household income, physical activity, smoking, alcohol consumption, history of cardiovascular disease, history of diabetes, and number of livebirths.

Analyses for spontaneous abortion, and stillbirth were additionally adjusted for number of spontaneous abortions, and stillbirths, as appropriate.

*p < 0.05.

**p < 0.01.

### Pregnancy Loss and All-Cause Mortality, Stratified by Age, as Well as the Presence of Cardiovascular Diseases or Diabetes

The associations between pregnancy loss and all-cause mortality stratified by age as well as the presence of cardiovascular disease or diabetes are presented in Table 3. Compared to women aged <50 without a history of pregnancy loss and cardiovascular disease, women aged ≥50 with a history of two or more pregnancy loss and cardiovascular disease had higher all-cause mortality: aHR 1.32, 95% CI 1.18–1.48. Women aged ≥50 with a history of two or more pregnancy loss and diabetes also had higher all-cause mortality: aHR 1.30, 95% CI 1.06–1.60.

**TABLE 3:** Cox proportional hazard ratios (95% confidence intervals) for all-cause mortality by pregnancy loss number, stratified by age as well as the presence of cardiovascular disease or diabetes (China, 2004–2008 for baseline characteristics and 2016 for all-cause mortality).

	The presence of cardiovascular disease					The presence of diabetes			
	Number of participants	Number of deaths	HR (95% CI)^a^	*p* for trend		Number of participants	Number of deaths	HR (95% CI)^b^	*p* for trend
Without cardiovascular disease & age <50					Without diabetes & age <50				
Total pregnancy loss				0.24					0.08
0	120,383	1,339	1			125,410	1,447	1	
1	10,266	138	1.05 (0.88, 1.25)			10,747	164	1.12 (0.95, 1.32)	
≥2	2,543	36	1.06 (0.76, 1.48)			2,688	42	1.10 (0.80, 1.49)	
Spontaneous abortion				0.71					0.17
0	123,689	1,388	1			128,879	1,499	1	
1	7,684	101	1.02 (0.83, 1.25)			8,036	125	1.14 (0.95, 1.37)	
≥2	1819	24	0.98 (0.65, 1.47)			1930	29	1.05 (0.72, 1.51)	
Stillbirth				0.10					0.25
0	129,437	1,457	1			134,908	1,595	1	
1	3,324	47	1.08 (0.81, 1.45)			3,485	48	0.98 (0.74, 1.31)	
≥2	431	9	1.51 (0.79, 2.92)			452	10	1.51 (0.81, 2.81)	
With cardiovascular disease & age ≥50					With diabetes & age ≥50				
Total pregnancy loss				<0.01					<0.01
0	28,735	2,885	1			7,163	1,112	1	
1	4,683	589	1.05 (0.96, 1.15)			1,102	213	1.15 (0.99, 1.33)	
≥2	1947	350	1.32 (1.18, 1.48)**			405	102	1.30 (1.06, 1.60)*	
Spontaneous abortion				<0.01					0.24
0	31,489	3,298	1			7,765	1,253	1	
1	3,033	380	1.00 (0.90, 1.11)			715	131	0.99 (0.82, 1.19)	
≥2	843	146	1.27 (1.07, 1.50)**			190	43	1.16 (0.85, 1.58)	
Stillbirth				<0.01					<0.01
0	32,090	3,315	1			7,954	1,258	1	
1	2,369	324	1.09 (0.97, 1.22)			549	118	1.24 (1.03, 1.51)*	
≥2	906	185	1.39 (1.20, 1.62)**			167	51	1.41 (1.06, 1.87)*	

a Adjusted for region, BMI, level of highest education, annual household income, physical activity, smoking, alcohol consumption, history of diabetes, and number of livebirths.

^b^ Adjusted for region, BMI, level of highest education, annual household income, physical activity, smoking, alcohol consumption, history of cardiovascular disease, and number of livebirths.

Analyses for spontaneous abortion, and stillbirth were additionally adjusted for number of spontaneous abortions, and stillbirths, as appropriate.

*p < 0.05.

**p < 0.01.

Women aged ≥50 with a history of two or more spontaneous abortion and cardiovascular disease had higher all-cause mortality: aHR 1.27, 95% CI 1.07–1.50. Similarly, women aged ≥50 with a history of two or more stillbirth and cardiovascular disease had higher all-cause mortality: aHR 1.39, 95% CI 1.20–1.62. Women aged ≥50 with a history of stillbirth and diabetes also had higher all-cause mortality: aHR 1.24, 95% CI 1.03–1.51 and 1.41, 95% CI 1.06–1.87 for one and two or more stillbirths, respectively.

Compared to women aged ≥50 with a history of pregnancy loss (total pregnancy loss, spontaneous abortion, and stillbirth) and cardiovascular disease, women aged <50 with a history of pregnancy loss (total pregnancy loss, spontaneous abortion, and stillbirth) and without cardiovascular disease were not significantly associated with all-cause mortality. The associations were also not significant for women aged <50 with a history of pregnancy loss (total pregnancy loss, spontaneous abortion, and stillbirth) and without diabetes.

### Pregnancy Loss and All-Cause Mortality, Stratified by Baseline Characteristics

**FIGURE 1:**
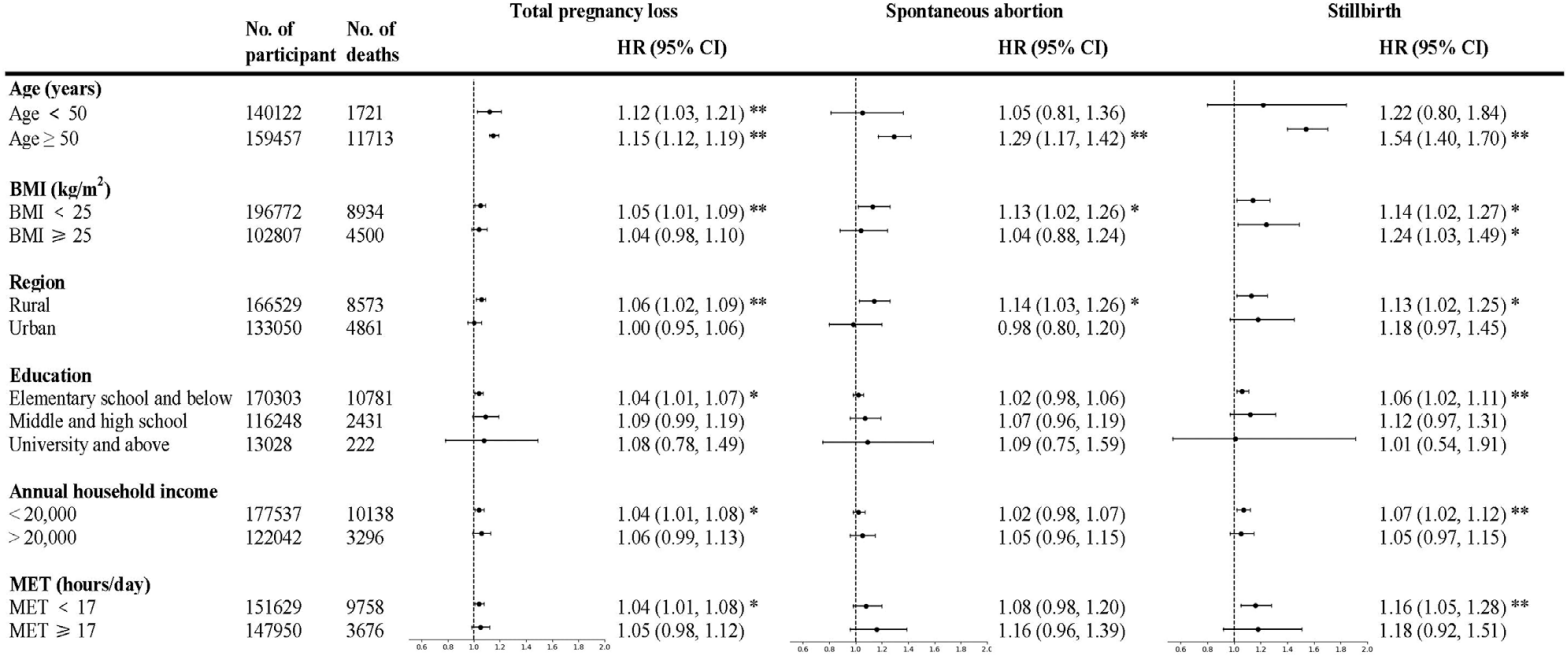
Cox proportional hazard ratios (95% confidence intervals) for all-cause mortality by pregnancy loss number, stratified by baseline characteristics (China, 2004–2008 for baseline characteristics and 2016 for all-cause mortality). Adjustments are as in Table 2 other than the stratified variable. Analyses for pregnancy loss, spontaneous abortion, and stillbirth are among women with at least one pregnancy loss, spontaneous abortion, and, stillbirth. BMI, body mass index; MET, metabolic equivalent task. *p < 0.05. **p < 0.01.

The associations between each additional pregnancy loss and all-cause mortality stratified by age, BMI, study region, educational level, annual income and MET hours are presented in Figure 1. Of statistical significance was the association between pregnancy loss and all-cause mortality in women between the ages <50 and ≥50 years old (<50 years old: aHR 1.12, 95% CI 1.03–1.21; ≥50 years old: aHR 1.15, 95% CI 1.12–1.19), whose BMI was less than 25 (aHR 1.05, 95% 1.01–1.09), who resided in rural regions (aHR 1.06, 95% CI 1.02–1.09), with educational levels elementary school and below (aHR 1.04, 95% 1.01–1.07), who had annual incomes of less than 10,000 yuan (aHR 1.04, 95% CI 1.01–1.08), and who had MET hours of less than 17 per day (aHR 1.04, 95% CI 1.01–1.08). However, the association between pregnancy loss and all-cause mortality in women whose BMI was ≥25, who resided in urban regions, who had educational levels middle and high school or university and above, who had annual income of more than 20,000 yuan, and who had MET hours ≥17 per day were not statistically significant.

The association between stillbirth with the risk of all-cause mortality in the subgroup analyses was broadly similar to that of pregnancy loss in general, while the association between spontaneous abortion with the risk of all-cause mortality in the subgroup analyses was largely non-significant.

## Discussion

In this cohort of Chinese women, pregnancy loss, in particular two or more spontaneous abortions and stillbirths, was associated with higher all-cause mortality. The associations between recurrent pregnancy losses and all-cause mortality were more pronounced in women aged above 50 with cardiovascular disease or diabetes.

Similar to our findings, a Danish register-based study on 1,001,266 women reported an increased risk of long-term mortality among women with a history of spontaneous abortion. The study also reported that each additional spontaneous abortion was associated with an increased risk of mortality (31). A recent finding from the Nurses’ Health Study II conducted in the United States on 101,681 women also reported an increased risk of premature mortality, particularly due to death from cardiovascular disease (32) in women with a history of spontaneous abortion. Furthermore, stillbirth and spontaneous abortion have been associated with APS (21,22,25), a condition in which the body produces antiphospholipid antibodies that binds to phospholipids or phospholipid-binding proteins within cell membranes, activating endothelial cells, monocytes, platelets, complements, and coagulation regulators that may induce fetal death (23,24). APS has been associated with venous thromboembolism, stroke, transient ischemic attack, heart valve disease, and coronary artery disease (23,24), which may further contribute to all-cause mortality. However, a cohort study on 54,652 Japanese women aged 40–79 years found that women with a history of two or more pregnancy losses had lower risk of mortality from cardiovascular disease compared to those with no history of pregnancy loss (16), while a cohort study on 267,400 Chinese female textile workers reported no significant associations observed between spontaneous abortion and stillbirth with cardiovascular disease-associated mortality (33). The reasons for the discrepant results may due to the differences in study population, sample size, study design, and differences in confounding factors adjusted across studies.

In this study, considering the effect of cardiovascular disease and diabetes on the association between pregnancy loss and all-cause-mortality, we performed subgroup analyses stratified by age and the presence of cardiovascular disease and diabetes. The result demonstrated the associations were more pronounced in older women with cardiovascular disease or diabetes at baseline. Our findings suggest that recurrent pregnancy loss may be an early marker for all-cause mortality in the presence of cardiovascular disease or diabetes among women aged above 50. Consistent with our findings, published research have indicated that women with a history of pregnancy loss are at greater risk of cardiovascular risk factors high blood pressure and type 2 diabetes later in a woman’s life (34), which is associated with higher risk of mortality (35). Given that metabolic processes deteriorate with age, the effect of age on the associations is unsurprising. Another study conducted using the CKB dataset also demonstrated that a history of pregnancy loss was associated with higher risk of developing cardiovascular disease in women later on in life, with a stronger relationship observed among women with recurrent pregnancy loss(13). A cohort study based on the European Prospective Investigation into Cancer and Nutrition (EPIC) demonstrated a 30% higher risk of new onset of diabetes in women with a history of spontaneous abortion, and a two-fold increased risk in those with recurrent spontaneous abortion (17). Similarly, two other studies in the Chinese populations reported similar findings (18,19). A study on 15,404 women found that stillbirth was significantly associated with a two-fold increased risk of future diabetes (20) while another reported that women with a history of ≥3 spontaneous abortions had 2.11 times higher risk of developing diabetes later on in life (18). Similarly, another study utilizing the CKB database reported similar findings, and that the HRs increased with increased number of pregnancy loss (19). Further prospective studies are warranted to longitudinally investigate whether pregnancy loss is associated with cardiovascular-related mortality or diabetes-related mortality.

Both high blood pressure and type 2 diabetes are known contributors to endothelial dysfunction (36). Endothelial dysfunction, a type of coronary artery disease, can contribute to both pregnancy loss and maternal mortality (37). Evidence suggests that maternal endothelial dysfunction prior to pregnancy may negatively affect embryo implantation and impair the function of the placenta during pregnancy, causing spontaneous abortion (37). In addition, endothelial dysfunction, initiates and promotes the progression of atherosclerosis, and can therefore, also contribute to the development of cardiovascular disease (38,39) and premature maternal mortality (40). Although the pathological mechanisms between pregnancy loss and diabetes remains unknown, the pathological mechanism is postulated to be similar as to cardiovascular diseases as suggested by current evidence (19,41,42). Hence, further research is warranted to better understand the underlying mechanisms linking pregnancy loss with all-cause mortality.

In our stratified analysis, we found that the association between pregnancy loss and all-cause mortality was more pronounced in older women, women who lived in rural areas, had lower MET hours, lower educational attainment, and/or lower household income. Findings were broadly similar in the direction and magnitude of the effects for the different types of pregnancy loss. This may be explained by the greater disease burden in the elderly, and the relatively lower healthcare quality in the rural area, which has also been associated with a higher risk of pregnancy complications and mortality (43). There is growing evidence that physical activity during pregnancy is beneficial for both the woman and fetus, and women who exercised during pregnancy have lower risk of pregnancy loss (44). In addition, previous studies have also reported that women with lower socioeconomic status and educational achievement displayed greater risk of pregnancy loss (45).

### Strengths and Limitations

This study is based on a large-scale prospective cohort which included women from 10 diverse regions of China, ensuring the generalizability of our findings to the Chinese population. The baseline survey also covered a comprehensive range of demographic, socioeconomic, and lifestyle information, allowing us to adjust for many confounders. In addition, data for this study was collected *via* standardized approaches with stringent quality control, making our results reliable and reproducible.

However, there are still several limitations that should be noted. First, the pregnancy history was self-reported, which may result in some certain recall bias. Second, although we have performed stratification analysis to identify associated factors, we were not able to include all the conditions or risk factors for pregnancy loss before or during pregnancies due to the lack of data. Third, although it is most likely that the majority of women developed diabetes after the age of reproduction, a small number of women included in our study may have gestational diabetes. Future research is needed to explore the potential effect of gestational diabetes on the association between pregnancy loss and all-cause mortality. Finally, residual confounding from other known or unknown risk factors may still exist despite a wide range of potential confounders taken into account for analysis.

### Conclusion

In conclusion, we found that recurrent spontaneous abortion and stillbirth, was associated with an increased risk of all-cause mortality in Chinese women, particularly among older women with cardiovascular disease or diabetes at baseline. Future studies are needed to further elucidate the mechanisms underlying these relationships to prevent the onset of long-term adverse health outcomes in women.
